# Validating a Multibiomarker Panel for the Assessment of Quantity and Quality of Plant Foods in the Diet (PLAENTI): Protocol for a Parallel Group–Designed Randomized Controlled Trial

**DOI:** 10.2196/77571

**Published:** 2026-02-27

**Authors:** Victor Schmalle, Julia Renz, Paola G Ferrario, Stephanie Seifert, Ann Katrin Engelbert, Oliver Wittek, Benedikt Merz, Lorraine Brennan, Claudine Manach, Manuela J Rist, Achim Bub

**Affiliations:** 1 Department of Physiology and Biochemistry of Nutrition, Max Rubner-Institut, Haid-und-Neu-Straße 9, Karlsruhe, 76131, Germany, +49 721 6625 ext 595; 2 Institute of Food and Health, School of Agriculture and Food Science, University College Dublin (UCD), Dublin, Ireland; 3 Human Nutrition Unit, INRAE, Université Clermont Auvergne, Clermont-Ferrand, France

**Keywords:** biomarkers of food intake, dietary assessment, multibiomarker panel, plant-based diet, randomized controlled trial, targeted metabolomics, standardized diet

## Abstract

**Background:**

Although a high intake of plant foods is often considered healthy, some plant foods can be detrimental to health. Reliable dietary assessment is crucial to examine the relationship between diet and disease. Current dietary assessment methods rely on self-reported intake data, which are subject to bias. Objective measurement using biomarkers of food intake could mitigate this problem. However, single biomarkers of food intake have limitations as well. Combining several biomarkers of food intake into a multibiomarker panel could attenuate these limitations and allow for an accurate, objective dietary assessment.

**Objective:**

The PLAENTI study aims to validate a multibiomarker panel for the assessment of quantity and quality of plant foods in the diet.

**Methods:**

PLAENTI is a randomized controlled trial with 4 arms in a parallel design. Metabolically healthy adults (≥18 years old) were enrolled in the study. The study consisted of 1 week of run-in, with a standardized diet low in healthful plant foods for all participants; 2 weeks of a dietary intervention according to the assigned arm; and 1 week of washout, during which participants returned to their habitual diet. During the intervention, the participants’ diet consisted of either a low, medium, or high proportion of healthful plant foods or a high proportion of unhealthful plant foods in the diet according to the assigned arm. The arm that received a high proportion of healthful plant foods served as the control. All food was provided based on energy-adjusted menu plans. During the visits, anthropometry and body composition were assessed, and blood samples were collected. Throughout the study, participants collected multiple urine samples (24-hour urine, evening and morning spot urine) and stool samples. Blood and urine samples will be analyzed by liquid chromatography-mass spectrometry to determine biomarker levels for the validation of a multibiomarker panel.

**Results:**

After receiving approval from the ethics committee, recruitment began, and the first screening visit took place in November 2023. Between January and August 2024, of the 66 enrolled participants, 59 (31 female, 28 male) successfully completed the study, and their urine, blood, and stool samples are available for analysis. PLAENTI was conducted in 5 waves with a maximum of 16 participants enrolled in each wave. The mean age of the study population was 45.5 (SD 18.4) years, the mean BMI was 24.8 (SD-3.9) kg/m², and the mean total energy expenditure was 2464 (SD 440) kcal.

**Conclusions:**

PLAENTI was conducted in a highly controlled and standardized manner, yielding samples and data that will be used to examine whether the quantity and quality of plant foods in the diet can be assessed using a multibiomarker panel. Successful validation of the multibiomarker panel would enable its application for objective dietary assessment.

## Introduction

A high intake of plant foods within a diet is often considered healthy. However, there are also plant foods that can be detrimental to health. For this reason, plant-based diet indices have been developed, which distinguish between “healthful” and “unhealthful” plant foods and form 3 categories: a general plant-based diet index, a healthful plant-based diet index, and an unhealthful plant-based diet index [1,2]. This distinction between healthful (eg, whole grains, unprocessed fruits and vegetables, nuts, and seeds) and unhealthful (eg, refined grains, sugar-sweetened beverages, and unfavorable vegetable oils) plant foods made it possible to obtain more detailed data on the relationship between diet and disease [1,2]. To date, nutritional epidemiology has relied on self-reported intake data, collected by tools such as food frequency questionnaires (FFQs), 24-hour recalls, and weighed or estimated diet records, which are prone to bias [3-6]. Self-reported data are subjective and influenced by factors such as the social desirability of food items, how individuals estimate portion sizes, the training of subjects, and the interview itself [3,7]. Well-known inaccuracies of plant food assessment include overreporting of fruit and vegetable intake and underreporting of oil and nut consumption [8-11]. The reliability of data may be enhanced by repeated measurements, improvement of assessment tools, or country-specific adaptation [7,12]. On the other hand, repeated measurements or higher-validity tools, such as 7-day dietary records, will increase the burden on participants [12].

There is growing evidence that biomarkers of food intake (BFIs) are a valuable tool for improving the accuracy of dietary assessment by introducing objective measurement [6,13-17]. However, BFIs also have limitations. There are overlaps between different foods with the same biomarker, and only a few food-specific biomarkers are known. Additionally, the concentration of some BFIs in biospecimens can vary between individuals, depending on factors such as age, sex, physical activity, and body composition [18]. Validation of candidate BFIs is of key importance and still scarce [15,19]. Combining several biomarkers into a multibiomarker panel (MBP) may mitigate the limitations of single BFIs and self-reported intake data [13,20]. It has been shown to increase the specificity and robustness of dietary assessment for individual foods (eg, banana, cocoa, and walnut) and for food groups such as total fruit intake [21-24]. However, dietary patterns, rather than individual nutrients or foods, have become a prominent topic in epidemiology, and several associations have been discovered between dietary patterns and noncommunicable diseases [25-28]. To apply BFIs and MBPs in epidemiological research, it is necessary to study and validate BFIs for the habitual diets of individuals or dietary patterns within populations. Accordingly, MBPs that include specific BFIs, BFIs for food groups, and BFIs that overlap between several food items could be promising to cover dietary patterns [29-34].

Cuparencu et al [35] recently reviewed validation criteria for BFIs and the general concepts of objective dietary assessment and listed known BFI candidates and their utility levels, as well as potential MBPs. The review also proposes research gaps. These include the identification of new MBPs and the discrimination of, for example, animal versus plant foods or healthful versus unhealthful foods. Additionally, more data on the interplay between BFI kinetics, frequency of food consumption, sampling schemes, sampling time windows, and different biospecimens are needed [35]. Furthermore, most studies still focus on biomarker discovery, often with untargeted analytical methods [33,36]. Beckmann et al [37] measured an MBP consisting of 54 BFI candidates in the urine samples of 15 participants who received a 3-day menu plan. The researchers were able to discriminate between the 3 dietary patterns that mimicked common UK diets [37]. Ultimately, more research is needed to unlock the full potential of BFIs and MBPs. Intervention studies specifically designed to measure BFIs in various samples from a diverse group of individuals consuming exact amounts and types of food, while considering dose- and time-response aspects, are necessary.

The JPI HDHL–funded project Plant*Intake* (acronym for “Combining biomarker panels and dietary intake data for improved assessment of healthful/unhealthful plant food intake”) aims to adapt the plant-based diet indices described by Satija et al [1] to better fit to European diets and common foods (European plant-based diet indices). Furthermore, an MBP reflecting the quantity and nutritional quality (healthful vs unhealthful) of plant foods in the diet will be derived using intake data and samples from previous studies. Validation of MBPs is paramount for future use. Therefore, the developed MBP will be validated with data from the intervention study PLAENTI (“Validating a multibiomarker panel for the assessment of quantity and quality of plant foods in the diet”), which is described in this study protocol.

The aim of the PLAENTI study is to validate an MBP for the assessment of the quantity and nutritional quality of plant foods in an omnivorous diet. Accordingly, the primary objective of the validation study PLAENTI is to test whether the quantity of plant foods within the diet can be correctly estimated by measuring levels of selected urinary and blood biomarkers that are part of the MBP. A secondary objective is to test whether a difference in nutritional quality (healthful vs unhealthful) of plant foods in the diet can also be detected with the MBP. The effect of potential confounders (age, sex, physical activity, and body composition) on the precision of estimating plant food intake using the MBP will be assessed. Finally, the MBP will be determined in both 24-hour urine and spot urine samples. This will allow a comparison of the different sample types (24-hour urine collection, evening and morning spot urine, and different pools of these spot urines) to assess their reliability in reflecting dietary patterns through biomarker analysis. To address these objectives, participants received a 2-week dietary intervention with different proportions and nutritional qualities of plant foods, while urine and blood samples were collected at several time points.

## Methods

### Study Design

The PLAENTI study was conducted at the Study Center for Human Nutrition of the Max Rubner-Institut, Federal Research Institute of Nutrition and Food in Karlsruhe, Germany, from January 9 to August 15, 2024. PLAENTI was designed as a randomized controlled trial with 4 arms. A parallel-group design was chosen to efficiently conduct the study and allow for direct comparison of results between the arms. A total of 66 participants were enrolled and randomized to 1 of 4 arms, stratified by sex. For logistic reasons, the study was conducted in 5 waves, each with a maximum of 16 participants. Each wave lasted a total of 4 weeks and started with a 1-week run-in period during which all participants consumed the same diet consisting of a low proportion of healthful plant foods to achieve comparable baseline levels of biomarkers. For the 2-week intervention period, participants were assigned to 1 of 4 interventions and received a diet that varied in the quantity and nutritional quality of plant foods to mimic different dietary patterns. The intervention was followed by a 1-week washout period, during which the participants returned to their habitual diet.

**Figure 1.**
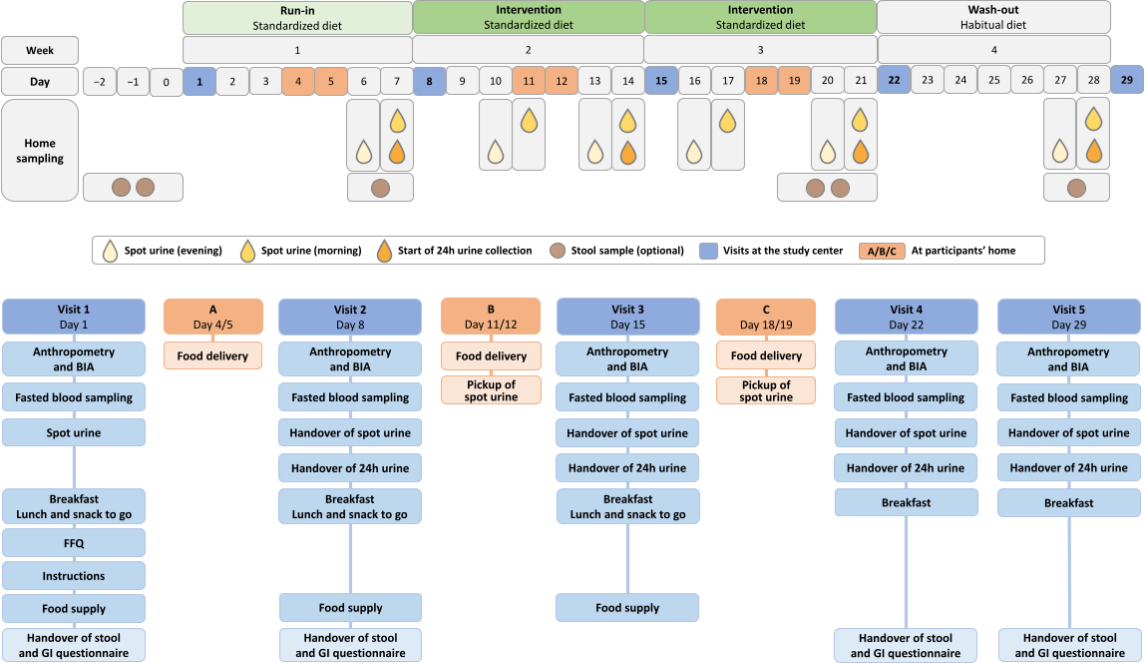
Overview of the study procedure. The figure shows the time sequence of run-in, intervention (either low, medium, or high in healthful or high in unhealthful plant foods), and washout, with corresponding visits to the study center and sample collections at home, as well as the schedule of visits to the study center and deliveries (A, B, and C). BIA: bioelectrical impedance analysis; FFQ: food frequency questionnaire; GI questionnaire: gastrointestinal symptom questionnaire.

The study was carried out in an outpatient setting. Participants collected urine and stool samples and prepared and consumed food at home. Visits to the study center took place once a week on an identical schedule for all participants. Participants were asked to fast for at least 10 hours beforehand and underwent a series of standardized examinations and measurements described in detail below. Standard operating procedures were developed and tested for feasibility for all interviews, examinations, measurements, sample collections, sample preparations, and storage. Interviewers and examiners were extensively trained before and supervised during data collection. An overview of the study procedure is shown in Figure 1.

Prior to PLAENTI, an internal feasibility study called prePLAENTI was conducted (unpublished; registered at the German Clinical Trials Register: DRKS00031484, available at [38]). The purpose of prePLAENTI was to evaluate the acceptability of the standardized diets for participants and the feasibility of the planned study procedures. Participant feedback and process evaluation by the study center were used to ensure that PLAENTI can be conducted efficiently.

### Recruitment

Female and male volunteers aged 18 years or older were eligible to participate. Detailed inclusion and exclusion criteria are listed in Textbox 1. Medical history was obtained using a health status questionnaire, and a basic physical examination was performed.

Textbox 1. **Eligibility criteria of the PLAENTI study*.***

**Inclusion criteria**

- Females and males, 18 years or older
- Volunteers who are able to comply with the study terms
- Parameters of liver and kidney function, carbohydrate and lipid metabolism, showing no pathological significance
- Nonsmokers
- Omnivorous diet
- Written informed consent to participate in the study

**Exclusion criteria**

- Diseases affecting the absorption, digestion, metabolism, or excretion of nutrients
- Acute or chronic infectious diseases
- Tumors requiring therapy
- History of bariatric surgery
- History of gastrointestinal tract surgery affecting the absorption, digestion, metabolism, or excretion of nutrients
- History of pancreas surgery
- Cholecystectomy
- Acute or regular intake of medications resulting in energy or nutrient malabsorption
- Allergy or intolerance to any food or food ingredient in the study diet
- Use of dietary supplements <2 weeks prior to the study period
- Use of antibiotics <3 months prior to the study period
- Blood donation during or <4 weeks prior to the study period
- Pregnancy and breastfeeding
- Illegal drug use
- Alcohol or drug abuse
- Participation in other clinical trials that may affect the outcome of this study
- Individuals living in a care facility
- Individuals deprived of their liberty by a court or administrative order
- Individuals who are not expected to comply with the terms of the study

Participants were recruited in Karlsruhe, Germany, and the surrounding area. Posters and flyers were distributed at local and regional universities and public institutions (eg, libraries), doctors’ offices, pharmacies, and health care facilities. In addition, recruitment was carried out via local/regional print media and social media in accordance with the requirements of the “Social Media Guideline” [39]. In addition, invitation emails were sent to potentially eligible participants listed in the study center’s participant database to inform them about the study. First, persons interested in study participation were informed about the nature and scope of the trial in a detailed telephone screening. Then, potential participants were asked to visit the study center for a screening that included basic physical examinations and a detailed information session.

### Allocation

The allocation sequence was generated by a biostatistician who was not involved in any further steps during the ongoing study and had no direct contact with the participants. Participants were randomly assigned to 1 of the 4 intervention arms with a 1:1:1:1 allocation by repeatedly using the “sample” function of the R software (R Core Team). Specifically, cluster randomization was used with clusters of different sizes. The clusters were heterosexual couples on the one hand and individual persons on the other. We randomized within waves for logistic reasons and stratified by sex to assess effects for females and males independently. The result of randomization was a sequential list of intervention arms with the corresponding study identifier of participants. The randomization plan is reproducible.

### Blinding

Due to the nature of the dietary intervention, neither the participants nor the research staff responsible for carrying out the intervention were blinded to the intervention allocation. Only the study staff responsible for data collection, sample processing, and analysis were blinded.

### Intervention

During the intervention, participants received an omnivorous diet that varied in the quantity and nutritional quality of plant foods according to their assigned arm: (1) low proportion of healthful plant foods (LH), (2) medium proportion of healthful plant foods (MH), (3) high proportion of healthful plant foods (HH), or (4) high proportion of unhealthful plant foods (HU). The intervention HH served as the control, as the diet implemented here comes closest to the intake of plant foods recommended by international dietary guidelines (eg, the “healthy reference diet” proposed by the EAT-*Lancet* Commission on Healthy Diets from Sustainable Food Systems [40]).

The intervention arms were designed to mimic dietary patterns similar to those described by the original plant-based diet indices [1]. To better reflect European eating habits, slight adjustments were made to the categorization of plant foods as healthful or unhealthful, and plant-based alternatives were included. Whole grains, fruits, vegetables, legumes, nuts, vegetable oils, tea, and coffee were categorized as healthful. Refined grains, fried potato products, fruit juices, sugar-sweetened beverages, sweets, and desserts were categorized as unhealthful. As literature on the nutritional quality of potatoes does not distinguish between different preparation methods, there is a lack of evidence to classify cooked, baked, or roasted potatoes as healthful or unhealthful. For this reason, this food group was categorized as “neutral.” Plant-based alternatives were also classified as neutral due to their heterogeneous nature and inconsistent evidence on health associations. Animal-based foods were also viewed as neutral, and no BFI candidates for animal food were considered for the intended biomarker analysis.

For this study, a total of 35 base menu plans with an average energy content of 2000 (SD 50) kcal were calculated with the software PRODI (version 7.2, Nutri-Science GmbH). These base menu plans were later energy-adjusted to create the menu plans for the participants as described in the following section. Each menu plan consisted of 4 meals. In total, 7 menu plans were calculated for the run-in period and 7 for each intervention arm. During both intervention weeks, the same 7 daily menu plans were used. However, the sequence of these menu plans differed between the first and the second week, resulting in a variation in the foods consumed in relation to the sampling times. This should help to test whether the MBP is robust and can correctly assign participants to their intervention based on the overall dietary pattern, rather than by single dishes.

The main metric used to calculate the menu plans was the proportion of plant food servings while adhering as closely as possible to a macronutrient ratio of 50/30/20 energy percent for carbohydrates/fat/protein. Table 1 shows the average proportions of plant foods in the final menu plans of the run-in period and the 4 arms during the intervention. The base menu plans and the definition of serving sizes can be found in Multimedia Appendix 1.

**Table 1.** Proportions of plant foods in the calculated menu plans. Proportions of plant foods (in %) in the diet of the run-in and the 4 arms during the intervention by the metrics of servings, weight, and calories.

Metrics	Run-in	LH^a^	MH^b^	HH^c^	HU^d^
Proportion of plant foods of total servings (in %)	39	43	62	84	82
Proportion of plant foods of total weight (in %)	51	47	61	86	79
Proportion of plant foods of total calories (in %)	50	50	67	87	85

a LH: low proportion of healthful plant foods.

b MH: medium proportion of healthful plant foods.

c HH: high proportion of healthful plant foods.

d HU: high proportion of unhealthful plant foods.

For each meal, a time window for consumption was defined: breakfast (6 AM to 10 AM), lunch (11:30 AM to 2:30 PM), snack (10 AM to 5 PM), and dinner (5 PM to 8:30 PM). Alcohol, extra sugar, and dietary supplements were prohibited, while coffee, black tea, and green tea were limited to a total of two cups (=300 mL) per day. All participants could freely consume 100 mL of cow milk daily to add to coffee or tea.

Prior to each of the 5 study waves, the base menu plans were individually energy-adjusted to the calculated total energy expenditure (TEE) of each participant (see the following section), and the corresponding recipes were created. If the given portion sizes were too large, the participants were allowed to reduce the amounts of ingredients evenly to maintain the proportion of plant foods. In this case, they had to weigh back the leftover food. For this purpose, all participants were provided with kitchen scales from the same manufacturer in order to ensure comparability. If the participants were still hungry, they could eat an additional snack consisting of white bread and cream cheese, with low impact on plant biomarkers. Any other modifications to the intervention were prohibited.

Participants prepared and consumed meals and collected urine and stool samples at home, carrying out the intervention as part of their daily life. For this purpose, the study center fully supplied them with all the necessary food in the required quantities and provided them with printed and digital recipe folders containing all the information, instructions, recipes, and daily logs. All recipes included a list of the individual energy-adjusted values of the ingredients and step-by-step instructions on how to cook or prepare the meals. The daily logs had to be completed during the run-in and the intervention. Here, participants were asked to record the exact time they ate each meal, the amount and type of tea or coffee they drank, and how much of the additional snack, if any, they consumed. There was also space for notes, mostly about permitted deviations from the recipes, such as the exact amount of food that was not eaten and weighed back.

Participants were closely monitored throughout the study. Personal contact was the main mode of communication, with 2 contacts per week (visits to the study center and food deliveries to the participants’ homes) as well as the possibility of contacting the study center by email on weekdays or the study hotline (SMS text message and phone call) at any time.

### Energy Adjustment

The menu plans were linearly energy-adjusted using Excel (Microsoft Corp) to create the individual menu plans for the participants. Total energy expenditure (TEE, in kcal) of each participant (see section Total Energy Expenditure Calculation) divided by the total kcal of the base menu plan yields a factor. This factor was then multiplied with the weight (in grams) of each food item in the base menu plan so that the plant proportions and macronutrient ratios are not altered. Resulting values ≤10 were mathematically rounded in increments of 1; values >10 in increments of 5; values >50 in increments of 10. The food items pretzel rolls (frozen), pretzels (frozen), schnitzel (vegan and pork, frozen), eggs, and crispbread were rounded to whole pieces. Wraps, bread rolls, and bagels were rounded to 0.5 piece increments.

### Food Logistics

Food purchase, preparation of food packages, and delivery to the participants were carried out based on the energy-adjusted menu plans by the study center staff. Food was purchased at wholesale markets and supermarkets. The selection of food items remained the same for each of the 5 waves and was not adapted to the seasons. Food items that are not available year-round, such as strawberries, were only used frozen.

All shelf-stable foods were delivered to participants before the first study day. Frozen foods were delivered once a week. Fruits, vegetables, and refrigerated foods (dairy and meat products) were supplied twice a week, at the visit to the study center and via home delivery.

### Anthropometry and Body Composition Assessment

Anthropometric data were assessed at all visits using standard methods. Measurements were taken in underwear, without shoes. Waist and hip circumferences were measured to the nearest 0.1 cm using the ergonomic measuring tape seca 201 (seca GmbH & Co KG). Three measurements were taken for each circumference, and the averages were recorded for analysis. Body composition was assessed by bioelectrical impedance analysis (BIA) using the seca mBCA 515. Body weight was measured to the nearest 0.05 kg, also using the seca mBCA 515, and height was measured to the nearest 0.01 m, using the seca 285 height measuring device. Weight and height were used to calculate the respective BMI (in kg/m²).

### Total Energy Expenditure Calculation

The TEE was calculated to enable adjustment of the menu plans to the individual energy requirements of each participant. To determine the TEE, resting energy expenditure (REE) and activity energy expenditure (AEE) were calculated. For REE calculation, the fat-free mass (FFM), which was determined as part of the BIA measurement, was used in the formula of Armbruster et al [41]:

REE (kcal) = 21.14 × FFM (kg) + 345.5

The AEE was determined using accelerometers (move 4, movisens GmbH). The accelerometers were placed on the participants’ hips at the screening visit and were then worn for 1 week. The software *SensorManager* (version 1.16.1, movisens GmbH) was used to program the sensor and read out the acquired data, and the software *DataAnalyzer* (version 1.15.1, movisens GmbH) was used to analyze the data and calculate the required parameters.

The participants’ TEE was calculated with the estimated REE, the AEE measured by accelerometry, and the diet-induced thermogenesis (DIT), which is assumed as 10% of the sum of REE and AEE [42]:

TEE (kcal) = REE (kcal) + AEE (kcal) + DIT (kcal)

### Habitual Diet Assessment (30-Day Food Frequency Questionnaire)

The validated 57-item FFQ applied in the German Health Interview and Examination Survey for Adults 2008-2011 (DEGS) of the Robert Koch-Institut was used to assess the habitual diet of the participants [9]. The FFQ includes questions about the frequency of consumption and usual portion sizes of different food groups consumed by the participants in the 4 weeks prior to study entry. The questionnaire was given and explained to the participants at visit 1 at the study center, with the request to complete it at home and return it at visit 2.

### Urine and Blood Samples for Biomarker Analysis

#### Analytical Method

During the study, several urine and blood samples were collected to measure BFIs by liquid chromatography-mass spectrometry (LC-MS). The LC-MS method is being developed independently of the PLAENTI study in the Plant*Intake* project. It aims to quantitatively analyze the BFIs described in the list of biomarkers that can be found in Multimedia Appendix 2. The samples of PLAENTI will be analyzed using the finalized analytical method.

#### Blood Samples

Blood samples were collected at every visit after at least 10 hours of fasting. Participants were placed on an examination couch in the supine position. After cutaneous disinfection, a tourniquet was moderately applied (safety blood collection device: Venofix Safety, B. Braun SE), and blood samples were taken from an antecubital vein by experienced medical study staff. Blood was drawn into ethylenediaminetetraacetic acid (EDTA)- or lithium-heparin-containing S-Monovette tubes (Sarstedt AG & Co KG) for plasma collection and into anticoagulant-containing serum-gel S-Monovette tubes (Sarstedt AG & Co KG) for serum collection.

After venipuncture, the filled tubes for plasma preparation were immediately centrifuged (20 °C, 10 minutes, 2500× *g*, Heraeus Multifuge X3R, Thermo Fisher Scientific) and kept on ice until aliquoted into microtubes. Erythrocytes (from lithium-heparin tubes) were washed with 0.9% sodium chloride solution and pipetted into CryoPure tubes (Sarstedt AG & Co KG). Tubes for serum preparation were allowed to clot for 30 minutes at room temperature, then centrifuged (20°C, 10 minutes, 2500×*g*) and kept on ice until aliquoted into microtubes or CryoPure tubes. Microtubes containing EDTA plasma, lithium-heparin-plasma, or serum were frozen at −20°C and then stored at −70°C until analysis. CryoPure tubes containing erythrocytes or serum for long-term storage were frozen at −20°C and then stored in the cryogenic storage tanks at −194°C.

#### Urine Samples

For urine sampling, participants received detailed oral instructions from trained study staff and a folder with written manuals for the collection procedures, including a calendar of sampling times. They were also given a log to record the exact time of sampling, any difficulties, and, for females, whether they were menstruating.

The 24-hour urine samples were collected from the morning before until the morning of visits 2, 3, 4, and 5. Participants were asked to start the urine collection after their first morning void, which was collected as a morning spot urine sample. Participants were provided with 3 urine collection containers (2 L capacity each, Sarstedt AG & Co KG) and cool bags with thermal packs. During collection, participants were asked to store their urine at home in the refrigerator or in a cool bag with the prechilled thermal packs to keep the collected urine permanently cool. This cool bag was also used to ensure the chilled transport of the collected urine to the study center on visit days. At the study center, the volume of the collected 24-hour urine was determined. The urine was mixed, and 15 mL of it was pipetted into a tube, centrifuged (4 °C, 10 minutes, 2500× *g*), and kept on ice until aliquoted into microtubes or CryoPure tubes. Microtubes containing 24-hour urine were frozen at −20°C and then stored at −70°C until analysis. CryoPure tubes for long-term storage of aliquots were frozen at −20°C and then stored in cryogenic storage tanks at −194°C.

Spot urine samples were collected from participants at home the evening and morning before the start of the 24-hour urine collection. Additional spot urine samples were collected halfway through each intervention week, also in the evening and the following morning (see Figure 1). The evening sample had to be the last urine before going to bed, and the morning sample had to be the first morning void. Spot urine samples were collected in 100 mL polypropylene collection cups with an integrated transfer unit in the screw caps and directly transferred to 10 mL vacuum tubes (V-Monovette, Sarstedt AG & Co KG). Participants were asked to store the filled vacuum tubes in the refrigerator immediately after sampling. A cool bag with prechilled thermal packs was used to transport the samples to the study center. At the study center, the vacuum tubes were centrifuged (4°C, 10 minutes, 2500× *g*) and kept on ice until aliquoted into microtubes or CryoPure tubes. Microtubes containing spot urine were frozen at −20°C and then stored at −70°C until analysis. CryoPure tubes for long-term storage of aliquots were frozen at −20°C and then stored in cryogenic storage tanks at −194°C.

### Stool Samples

The collection of stool samples was optional for participants. Their willingness to provide a stool sample was queried at the screening visit. Participants who agreed to provide stool samples were given detailed oral instructions and a written manual on the sampling procedure and were provided with stool collection kits containing a fecal sample collection aid (servoprax GmbH), a collection cup (Sarstedt AG & Co KG), a disposable spatula, and a 60 mL twist-seal bag (VWR International GmbH), as well as a thermal pack for transport, gloves, and zip-log bags for packaging. Participants collected two cherry-sized stool samples (approximately 5 g) on different days in a 72-hour window before visit 1 and visit 4, and one cherry-sized stool sample in a 48-hour window before visit 2 and visit 5. Samples were frozen at −20°C immediately after collection at the participants’ home, delivered frozen to the study center, and then stored at −70 °C until aliquoted. Samples were thawed and manually mixed inside the collection bags before taking aliquots of approximately 500 mg each for further processing. Aliquots were frozen immediately at −70°C until further analysis.

Participants who collected stool samples also completed a short questionnaire on gastrointestinal symptoms at visits 1, 2, 4, and 5, concerning the previous week or 2 weeks, respectively. The questionnaire covers stool frequency as well as the occurrence and frequency of the following symptoms: abdominal pain, heartburn, burping, watery stool or diarrhea, lumpy or hard stool, constipation, feeling full after food intake, gastrointestinal sounds, flatulence, nausea, and increased or decreased hunger or appetite.

Stool samples were collected for exploratory analyses. First, we can examine the effects of different quantities and qualities of plant foods on the gut microbiota. Another approach that can complement PLAENTI is to detect plant taxa in stool samples by genetic analysis, as described by Petrone et al [43].

### Compliance

Care was taken to ensure that only the most conscientious individuals were included in an attempt to keep the dropout rate as low as possible. During the telephone screening and the information session at the screening visit, potential participants were given detailed information about the study procedure and, in particular, the effort required to take part in the study. In addition to the inclusion and exclusion criteria, potential participants were asked about other issues such as food aversions, usual eating habits, and the compatibility of their daily routine with participation.

To improve participants’ compliance during the ongoing study, a number of measures were taken. As detailed in the “Intervention” subsection, food consumption was monitored using daily logs. Participants had to record their exact mealtimes, as well as the exact amount of uneaten food, in these daily logs. There was also a urine collection log to document the time of sampling. There was very close personal contact between the study staff and the participants during the weekly visits and food deliveries. In addition, the team of the study center was always available by email or telephone to directly respond to any questions or concerns that participants might have and to provide them with optimum support. Noncompliance occurred when relevant samples were missing, instructions regarding the intervention were not followed, or medical events (medication, illness) conflicted with participation. Noncompliance led to exclusion.

### Adverse Events

Safety monitoring included the recording, assessment, and follow-up of adverse events and the determination of the following safety parameters in blood at the first and last visit: gamma glutamyl transferase, glutamic oxaloacetic transaminase, bilirubin (total), creatinine, uric acid, glucose, triglycerides, and total cholesterol (all in serum), and a blood count. Only commercially available food was used, which was fully provided by the study center. Allergies to any food or food ingredient in the study diet were assessed in advance and resulted in exclusion (see Textbox 1). Therefore, no serious adverse events related to the consumption of the intervention diet were expected. Participants with unacceptable adverse events or acute illnesses that interfered with study participation were excluded from the study. This decision was made individually by the study physicians.

### Statistics

#### Outcomes

The primary outcome of the study is an MBP measured in urine and blood samples from participants in the arms LH, MH, and HH at defined time points during the 2-week intervention period. The analysis metric for the primary outcome is the change in levels of biomarkers contained in the MBP from visit 2 to visit 4, considered as a combined panel value. The variables will be aggregated and described by average values and SDs.

The secondary outcome is the MBP measured in urine and blood samples at defined time points in the two arms, HH and HU. The analysis metric for the secondary outcome is also the change in levels of biomarkers contained in the MBP from visit 2 to visit 4, considered as a combined panel value. The variables will be aggregated and described by average values and SDs.

Moreover, the comparability of the biomarker levels in the different types of urine samples (24-hour urine, spot urine, and pooled spot urine) will be investigated to gain insight into the applicability of spot urine sampling in epidemiological studies. Other outcomes of an exploratory nature will also be measured. Metabolomic analyses will be performed by measuring different classes of metabolites in the urine and blood samples. For microbiome parameters, the analysis metrics are alpha diversity (number and uniformity of species distribution within a microbiome), beta diversity (dissimilarity of the composition of different microbiomes), and abundance of individual taxa in different microbiomes. The results of the FFQ will be used to characterize the participants’ habitual diet.

#### Sample Size

The MBP to be validated in this study will be developed at a later stage of the Plant*Intake* project, so it was not possible to calculate the sample size prior to submission of the project proposal. As a Williams test is performed in the main statistical analysis, this was used as the basis for a sensitivity analysis. The calculation was performed using the R package “MCPAN” (R version 4.1.0, R Core Team). The option “Type: Williams” was used. Specifically, for a 3-arm design (for the primary outcome) with n=15 per arm and expected values for the MBP of 1 (LH), 2 (MH), and 3.8 (HH), and a homoscedastic variance of 2.5, a power of 0.81 was obtained [21,44-46]. A total of 60 participants were expected to complete the study, with even distribution between the groups.

Based on experience with previous studies, including a study with low, medium, and high intake of fruits and vegetables by Watzl et al [47] and the more recent JPI FoodBAll study (Universal Trial Number: U1111-1177-1536), a dropout rate of 12% was assumed. Therefore, in order to analyze a total of 60 participants, the goal was to enroll 68 participants in total.

#### Statistical Methods

The aim of the primary statistical analysis is to detect a trend in the MBP as a function of the proportion of healthful plant foods consumed by the participants. Here, the values of the MBP determined at visit 4 are adjusted with the baseline values from visit 2. The MBP values are compared between the interventions using the Williams test. The comparison is made for the 3 diets with different proportions of healthful plant foods (low, medium, or high). For simplicity, it is assumed that the primary outcome is normally distributed and has a homogeneous variance. The statistical hypothesis is that the primary outcome will follow a trend for the ordered categories of low, medium, and high intake of healthful plant foods. Group HH, with a high proportion of healthful plant foods in the diet, serves as a control. The following 3 average values of the MBP are assumed for the 3 intervention groups: *μ*1*, μ*2*,* and *μ*3. All intervention groups have a comparable sample size. An average different response is expected in the group with low and medium consumption compared to the high consumption (control) of healthful plant foods. The global null hypothesis is formulated as *H*0*: μ*1*=μ*2*=μ*3 versus the alternative hypothesis *H*1*: μ*3*≥μ*2*≥μ*1, with at least one strict inequality. When modeling the MBP, adjustment is made for the following potential confounding variables: age, sex, body composition (assessed by BIA), and physical activity (assessed by accelerometry). The MBP is considered to be validated if a significant monotonic trend is observed (*P*<.05).

As the main secondary analysis, mean MBP values will be compared between the HH and HU intervention arms, characterized by similar proportions of plant foods but differing in nutritional quality (healthful vs unhealthful). Group differences will first be assessed using an independent-samples *t* test, followed by linear regression models incorporating the same set of adjusting variables.

As a further aim, biomarker levels will be compared between different urine samples (24-hour urine, single spot urine, and pooled spot urine). Temporal variability during the 2-week intervention will be characterized descriptively and by variance components. For this purpose, mixed-effects linear models with biomarker levels as the dependent variable, urine sample type and time as fixed effects, and participants as random effects will be applied. From these models, within- and between-participant variance and intraclass correlation coefficients will be estimated. Additional exploratory analyses can be conducted on metabolomic profiles, metabolic pathways, and further physiological or health-related variables. These outcomes will be analyzed using linear regression models. To account for multiple testing in high-dimensional metabolomics, *P* values will be adjusted using the Benjamini-Hochberg procedure.

In the case of missing data, the first step is to investigate what type of missing data occurs. If biomarker levels are below a reliable detection limit, these are modeled as “censored data.” A distinction will also be made as to whether the missing data are random or not, and they will be treated accordingly. We will not include the data of participants in subsequent analyses if an unacceptable adverse event occurred or if they were noncompliant.

### Data Management

The consortium partners have developed a data management plan that is updated regularly. Data will be published in accordance with the Code of Conduct of the German Research Foundation (DFG) “Guidelines for Safeguarding Good Research Practice” [48] and regardless of the magnitude and direction of the effects. We aim to publish and disseminate data and associated metadata in anonymized form according to the FAIR (Findability, Accessibility, Interoperability, and Reuse) guidelines.

### Ethical Considerations

The study was conducted in accordance with the Declaration of Helsinki. The described study procedures are in accordance with the protocol submitted to the ethics committee prior to recruitment. It was registered in the German Clinical Trials Register (DRKS00032738; available at [49]) and approved by the Ethics Committee of the State Medical Chamber of Baden-Württemberg, Stuttgart, Germany, in September 2023 (F-2023-085). The Universal Trial Number is U1111-1298-1562.

Consent to participate in the study, the processing of personal data, and the analysis of pseudonymized research data were documented by signing the informed consent form. A copy of the signed consent form was kept by the study management. The consent form was approved by the ethics committee. The participants received €50 (approximately US $59.5) as compensation for their time and effort.

Personal information about potential and enrolled participants is subject to medical confidentiality. The Max Rubner-Institut adheres to the rules of the State Medical Chamber, the General Data Protection Regulation, and the Federal Data Protection Act when processing data. Participant data and sample material will only be passed on to directly involved project partners in pseudonymized form. A list of the names assigned to study identifiers is stored separately at the study center and is subject to technical and organizational measures to ensure that the personal data cannot be assigned to the study participant by unauthorized persons. Thereby, only those responsible at the trial site have access to the personal data.

## Results

### Study Timeline

**Figure 2.**
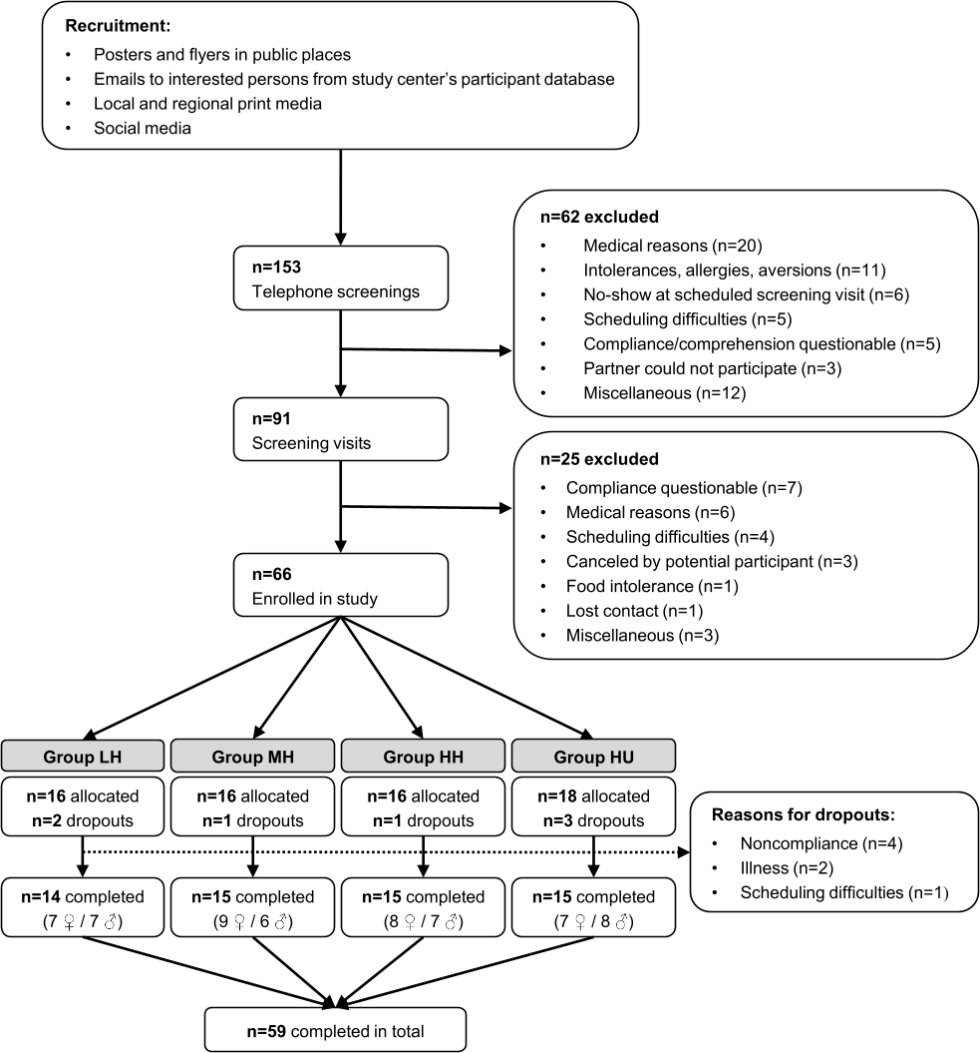
Participant flow. This flowchart shows the retention of participants from recruitment to completion of the study, including reasons for exclusion in all steps. Furthermore, the numbers of female and male participants of those who successfully completed the study are given. HH: high proportion of healthful plant foods; HU: high proportion of unhealthful plant foods; LH: low proportion of healthful plant foods; MH: medium proportion of healthful plant foods.

PLAENTI was conducted in 5 waves, from January 9 (first participant, first visit) to August 15 (last participant, last visit), 2024, each with a maximum of 16 participants, covering each intervention arm. After the final approval from the ethics committee was received on September 14, 2023, recruitment of potential participants was started on November 3, 2023, and the first screening visit took place on November 20, 2023. As the study was conducted in waves, participants were recruited continuously until the last wave began. As part of the recruitment process, a total of 153 telephone screenings were conducted with interested individuals. A total of 91 potential participants attended the screening visit, of which 66 participants were enrolled in the study. Five of them took part in the first study wave (January 9 to February 8), 14 in the second (February 20 to March 21), 15 in the third (April 9 to May 8), 16 in the fourth (June 4 to July 4), and 16 in the fifth wave (July 15 to August 15). In total, 7 participants (5 males and 2 females) were excluded after allocation for various reasons. Accordingly, 59 participants successfully completed the PLAENTI study, and their blood, urine, and stool samples are available for analysis. Detailed information about the participant flow is shown in Figure 2.

### Study Population

Baseline characteristics of the study population are given in Table 2. The calculated mean REE of the participants was 1679 (SD 278) kcal, the measured mean AEE was 785 (SD 205) kcal, and the calculated mean TEE was 2464 (SD 440) kcal. The proportion of female and male participants was 52.5% and 47.5%, respectively.

**Table 2.** Baseline characteristics of the study population. Number of females and males as well as characteristics on the first study day for the total study population and the intervention arms.

Characteristics	Total (N=59)	LH^a^ (n=14)	MH^b^ (n=15)	HH^c^ (n=15)	HU^d^ (n=15)
Female, n	31	7	9	8	7
Male, n	28	7	6	7	8
Age (years), mean (SD)	45.5 (18.4)	50.9 (19.3)	46.9 (20.1)	42.6 (17.6)	42.0 (14.8)
Age (years), range	19‐86	19‐76	21‐86	22‐69	20‐67
BMI (kg/m²), mean (SD)	24.8 (3.9)	25.7 (3.2)	24.0 (3.8)	24.2 (4.3)	25.3 (3.9)
TEE^e^ (kcal), mean (SD)	2464 (440)	2402 (510)	2312 (338)	2551 (517)	2572 (272)

a LH: low proportion of healthful plant foods.

b MH: medium proportion of healthful plant foods.

c HH: high proportion of healthful plant foods.

d HU: high proportion of unhealthful plant foods.

e TEE: total energy expenditure.

### Dietary Intervention

The calculation of menu plans resulted in a macronutrient proportion and fiber content of the diets as shown in Table 3.

**Table 3.** Macronutrient proportions and fiber content of the diets in the run-in period and the 4 intervention arms. The data shown are average values of the macronutrients and the fiber content of the menu plans calculated for the run-in and each arm.

Metrics	Run-in	LH^a^	MH^b^	HH^c^	HU^d^
Carbohydrates (in % of energy)	41	40	46	49	55
Fat (in % of energy)	35	35	33	34	31
Protein (in % of energy)	24	25	21	17	14
Fiber (total value in grams)	27	24	32	42	18

a LH: low proportion of healthful plant foods.

b MH: medium proportion of healthful plant foods.

c HH: high proportion of healthful plant foods.

d HU: high proportion of unhealthful plant foods.

## Discussion

### Addressing Research Gaps

Thanks to its carefully conceived experimental design, the PLAENTI study will be able to validate an MBP that reflects the quantity and quality of plant foods within omnivorous diets. Additionally, the study can address various aspects of BFI research, including several of the described validation criteria for BFIs. For example, aspects of dose-response can be assessed by comparing the 3 intervention arms with different quantities of healthful plant foods in the diet. Aspects of time-response can be explored by comparing biomarker levels at several time points during the intervention. Since data on physical activity, TEE, age, sex, and body composition were recorded, the effect of known confounders on BFIs can be considered. Furthermore, collecting 24-hour and spot urine samples, as well as blood samples, allows for a direct comparison of the informative value of data and burden on study participants of different sample types. The research gaps that PLAENTI can address align closely with the roadmap for using BFIs as assessment tools proposed by Cuparencu et al [35].

### Study Design

A crossover design may be considered advantageous for validation studies due to the reduced interindividual variability [50]. On the downside, a crossover design would significantly increase the duration of a standardized diet, which may result in higher dropout rates and a reduced likelihood of good compliance [51]. Because of this, we chose a parallel-group design in PLAENTI, even though this might lead to differences in baseline biomarker levels or confounders between participants. However, the run-in period will have reduced these baseline variations. The 2-week intervention is expected to be sufficient to establish a stable biomarker pattern, as the sampling windows for BFIs with long elimination half-lives are approximately 6 days [35].

We decided not to adjust macronutrient or fiber content, since our aim was not to investigate the health effects of the intervention, but instead we intended to mimic realistic dietary patterns. This will also increase the acceptability of the dietary intervention. The macronutrient proportions, as shown in Table 3, varied between the intervention arms, as expected. There are also differences in fiber content between arms, particularly between the HH and HU arms. This is naturally due to unhealthful plant foods having lower fiber contents than the healthful plant foods. To make the dietary intervention more acceptable in the HU arm, up to 10% of total servings were allocated to fresh fruits and vegetables instead of just unhealthful plant foods. This decision was based on the findings of the feasibility study prePLAENTI, which showed that participants found an extreme diet containing no healthful plant foods less acceptable (data not published; registered at the German Clinical Trial Register: DRKS00031484). Additionally, we prioritized fruits and vegetables that are commonly consumed and well accepted in the European Union. While this approach may limit overall diversity, it increases compliance and helps to obtain robust results for these plant foods. Eventually, we want to validate whether the MBP developed in the Plant*Intake* project can reflect common European diets. Overall, the study design of PLAENTI should be feasible for many research facilities. Ultimately, more whole-supply studies are needed to advance nutrition research. Exact intake data in the context of a complex diet allow us to consider matrix effects and biological relevance to obtain the most robust results regarding nutrition, foods, and diets. By providing this extensive protocol, we aim to facilitate the implementation of whole-supply studies by others, thereby advancing the quality and standard of nutritional interventions.

### Comparison to Prior Work

In related research, participants are often recruited from previous studies [16,34] or characterized by specific traits. For example, previous studies have only included postmenopausal females [36] or individuals with untreated prehypertension [52]. In other cases, samples and intake data from past cohorts are used [33]. In PLAENTI, we tried to best represent the general population to allow for a reliable validation of the MBP. To achieve this, we stratified by sex and included a wide age range.

Another important consideration is the accurate determination of the TEE. This ensures that participants receive the appropriate amount of food that meets their caloric needs. Ideally, the TEE would be determined directly using doubly labeled water, as Playdon et al [36] did. However, this is a time-consuming and expensive method, and therefore, we calculated it using REE and AEE instead. For the calculation of the REE, we used the formula of Armbruster et al [41], which was developed at the Max Rubner-Institut, and its applicability has been demonstrated [41]. The AEE was determined individually by accelerometry to provide more precise results. This is particularly important given the expected wide range of AEE levels, such as between older individuals and highly active young individuals. Often, the AEE is estimated by using the physical activity level (PAL). For example, Oude Griep et al [52] used a fixed PAL of 1.4 for all participants, which is comparable to the mean TEE/REE ratio of 1.47 of the individualized values in PLAENTI. Although our method did not allow for an exact determination, it provided a very good estimate of TEE. Just as there are different possible approaches to determining TEE, there are also different approaches to adjust menu plans to TEE values. One common approach is to use menu plans with predefined energy level increments and match participants to the plan that most closely aligns with their individual caloric needs [52-54]. Our individual approach is more accurate with the downside of increased logistical effort.

In a study design with a free-living population, compliance is one of the most critical factors, and a multitude of considerations must be made. In PLAENTI, we did not measure compliance objectively, but we implemented several methods to improve it. The most significant measure is the accurate supply and delivery of all required foods. The aforementioned manuals and schedules will have improved compliance, and the logs deliver a trust-based control. It was important to consider where burden was necessary for accurate results while making study participation feasible and acceptable. Measures described in the literature to facilitate compliance include providing 90% of TEE through strict menu plans and 10% from a restricted free-choice food list [52], prescribing or allowing a certain amount of specific foods within a habitual diet [55], or calculating menu plans based on each participant’s habitual diet to provide an individualized controlled version of it [36]. In PLAENTI, due to the validation purpose of the study, we chose a rather strict approach, where all foods in the menu plans were mandatory. Based on participant feedback from prePLAENTI, we permitted a total of up to two cups of coffee, black tea, or green tea per day to allow for some flexibility. Alcohol and dietary supplements were prohibited. The approach of allowing limited flexibility for coffee consumption was also used by Oude Griep et al [52], while they and Playdon et al [36] also allowed some leeway for alcohol consumption. The unique design of PLAENTI offers new methods and considerations for implementation in future whole-supply studies.

### Strengths

One of the major strengths of the PLAENTI study is that the study team purchased and distributed all of the food, thereby standardizing the diet. Although this was time-consuming and labor-intensive, it offered numerous advantages. First, participants were provided with the exact amount of food they needed. This helps reduce food waste, increase adherence to energy-adjusted menu plans, and prevent overconsumption. Furthermore, food safety (eg, cold chain) and quality could be ensured. In the study of Oude Griep et al [52], food was ordered online and delivered directly to the participants by grocers. While this approach is easier to implement, the study team loses precise control over food quality and is unable to split units of certain food items. In contrast, Playdon et al [36] provided precooked, portioned, weighed, refrigerated, or frozen food 3 times per week. Any unconsumed food was returned. While this approach is highly standardized and the best for ensuring compliance, it also requires the most effort, as well as appropriate cooking facilities and staff capacity.

Another strength of PLAENTI is the collection of different biological samples (24-hour urine, spot urine, and blood) at multiple time points. This increases the likelihood of covering a wide variety of BFIs, including those with short elimination half-lives, and allows for comparison of the informative values of different sample types. Furthermore, recording meal consumption and sampling times makes it possible to examine the temporal relationship between food intake and sample collection in the context of the analytical detection of BFIs. Several publications suggest that the applicability of spot urine samples may be comparable to 24-hour urine collections, and that both may be superior to blood samples [37,56,57]. Spot urine has the advantage of reduced burden, cost, and logistical effort compared to 24-hour urine collections [37,56]. We used vacuum tubes for spot urine sample collection to ensure optimal stability of the compounds [37]. The system was easy to use and allowed hygienic collection by the participants. Overall, PLAENTI may provide evidence for the use of spot urine samples as an alternative to 24-hour urine collections in BFI research. This could enable the widespread use of BFIs in nutrition-related studies.

### Limitations

One limitation of the PLAENTI study is that selection bias may have been introduced by the requirement that participants live within a reasonable driving distance (up to 40 km) of the study center for food deliveries. Additionally, the topic of the study may have particularly attracted individuals interested in nutrition. According to the eligibility criteria, participants were nonsmokers and metabolically healthy, which likely created a bias toward healthier lifestyles. This may also be reflected in the lower average BMI (24.8 kg/m²) of the study population compared to the general population of Germany (26.0 kg/m²) [58]. In this regard, the study population may differ from the general population. However, we do not expect this to significantly impact the results of the study.

Another limitation is that the composition of food varies, and secondary plant metabolites of fruits and vegetables are particularly affected [59-61]. This is also evident from the fact that metabolite concentrations can vary significantly even among apples from the same tree [62]. Since PLAENTI was divided into 5 waves from January to August, seasonal variations of the food metabolome may affect the results obtained. However, this also allows testing whether the MBP is robust under real conditions. By covering all intervention arms in each study wave, the effect of food seasonality on the comparability of results should be reduced [51]. To minimize the influence of other factors on food composition, food was purchased from the same grocers. Through access to the wholesale market, fruits and vegetables were mostly purchased in units from a single farm, which should reduce differences in the food metabolome. By providing detailed recipes (including preparation method, cooking time, and heat settings) and supplying food twice a week, we expect to have minimized the effects of different storage and processing. As not all foods are permanently available over an 8-month period, we replaced unavailable foods with the most comparable alternative. This mainly concerned processed animal products, which were not the focus of this trial.

### Conclusion and Prospects

A total of 59 participants successfully completed the PLAENTI study, and their urine, blood, and stool samples are available for analysis to validate the MBP. The study was conducted in a highly standardized setting to validate the MBP in terms of robustness, dose-response, sampling time, and reproducibility. Successful validation of the MBP would enable its application as a tool for objective dietary assessment in epidemiology. This, in turn, could contribute to a better understanding of the relationship between diet and disease.

## Supplementary material

10.2196/77571 Multimedia Appendix 1 Menu plans.

10.2196/77571 Multimedia Appendix 2 Biomarker list.

10.2196/77571 Checklist 1 SPIRIT checklist.

## Abbreviations

AEE: activity energy expenditure
BFI: biomarker of food intake
BIA: bioelectrical impedance analysis
DIT: diet-induced thermogenesis
EDTA: ethylenediaminetetraacetic acid
FFM: fat-free mass
FFQ: food frequency questionnaire
HH: high proportion of healthful plant foods
HU: high proportion of unhealthful plant foods
LC-MS: liquid chromatography-mass spectrometry
LH: low proportion of healthful plant foods
MBP: multibiomarker panel
MH: medium proportion of healthful plant foods
PAL: physical activity level
REE: resting energy expenditure
TEE: total energy expenditure
